# Increased Risk of Infection with Severe Fever with Thrombocytopenia Virus among Animal Populations on Tsushima Island, Japan, Including an Endangered Species, Tsushima Leopard Cats

**DOI:** 10.3390/v14122631

**Published:** 2022-11-25

**Authors:** Aya Matsuu, Kandai Doi, Keita Ishijima, Kango Tatemoto, Yushi Koshida, Ayako Yoshida, Kohei Kiname, Akio Iwashita, Shin-ichi Hayama, Ken Maeda

**Affiliations:** 1 Department of Veterinary Science, National Institute of Infectious Diseases, 1-23-1 Toyama, Shinjuku-ku, Tokyo 162-8640, Japan; 2 Transboundary Animal Diseases Research Center, Joint Faculty of Veterinary Medicine, Kagoshima University, 1-21-24 Korimoto, Kagoshima 890-0065, Japan; 3 Laboratory of Wildlife Medicine, School of Veterinary Medicine, Nippon Veterinary and Life Science University, 1-7-1 Kyonancho, Musashino, Tokyo 180-8602, Japan; 4 Department of Wildlife Biology, Forestry and Forest Products Research Institute, 1 Matsunosato, Tsukuba 305-8687, Japan; 5 Conservation and Animal Welfare Trust, Tsushima, 642-2 Kamiagata, Tsushima, Nagasaki 817-1602, Japan; 6 Center for Animal Disease Control, Kibana Campus, University of Miyazaki, 1-1 Gakuen-kibanadai-nishi, Miyazaki 889-2192, Japan; 7 Tsushima Rangers Office, Ministry of Environment, 1249 Izuhara, Tsushima, Nagasaki 817-0154, Japan

**Keywords:** SFTS, Tsushima leopard cat, wildlife, zoonosis

## Abstract

To investigate the seroprevalence of severe fever with thrombocytopenia syndrome (SFTS) among wild and companion animals on Tsushima Island, Japan, SFTS virus (SFTSV)-specific ELISA and virus-neutralizing tests were conducted on 50 wild boars, 71 Sika deer, 84 dogs, 323 domestic cats, and 6 Tsushima leopard cats. In total, 1 wild boar (1.8%), 2 dogs (2.4%), 7 domestic cats (2.2%), and 1 Tsushima leopard cat (16.7%) were positive for anti-SFTSV antibodies. Among the 11 positive animals, 10 were collected after 2019, and all were found on the southern part of the island. SFTSV, thus far, seems to be circulating within a limited area of Tsushima Island. To protect humans and animals, including endangered Tsushima leopard cats, from SFTSV infection, countermeasures are needed to prevent the spread of SFTSV on Tsushima Island.

## 1. Introduction

Severe fever with thrombocytopenia syndrome (SFTS) is an emerging tick-borne zoonotic disease caused by the SFTS virus (SFTSV), which is a negative-sense, single-stranded RNA virus in the *Bandavirus* genus of the *Phenuiviridae* family [1]. SFTS is endemic in countries of eastern and southeastern Asia [2,3,4,5]. The first human case of SFTS in Japan was diagnosed in 2012 [4], and since then, reports of SFTS cases have continued to increase. As SFTS is a viral hemorrhagic fever, patients show a variety of symptoms, including fever, vomiting, diarrhea, stomachache, hemorrhagic symptoms, and in severe cases, unconsciousness. The case fatality rates are 27% and 31% in Japan [6], 20% and 32.2% in South Korea [6,7], and 5% and 16.2% in China [8,9]. Symptomatic SFTS has also been reported in animals in Japan, including captive cheetahs [10], dogs [11], and cats [12,13]. The clinical features of animal SFTS in Japan are similar to those of human disease, but the fatality rate is higher in cats (58–62.5%) [12,13] and dogs (43%) [11] than in humans. Recent studies have reported the transmission of SFTS from symptomatic companion animals to pet owners or veterinary service personnel [14,15,16].

SFTSV exhibits a wide host range, and high seroprevalence of SFTSV antibodies have been reported in Sika deer (*Cervus nippon*) (10–43.2%) and wild boars (*Sus scrofa*) (8.6–53.6%) in endemic areas, including Yamaguchi [17], Kagoshima [18], Miyazaki [19], Ehime [20], and Nagasaki Prefectures [12]. The seropositivity rate in wild animals tends to be higher in endemic areas where human cases of SFTS have been reported, and thus, wild animals are believed to be superior sentinels for assessing the risk of SFTSV infection in endemic areas [17].

**Figure 1:**
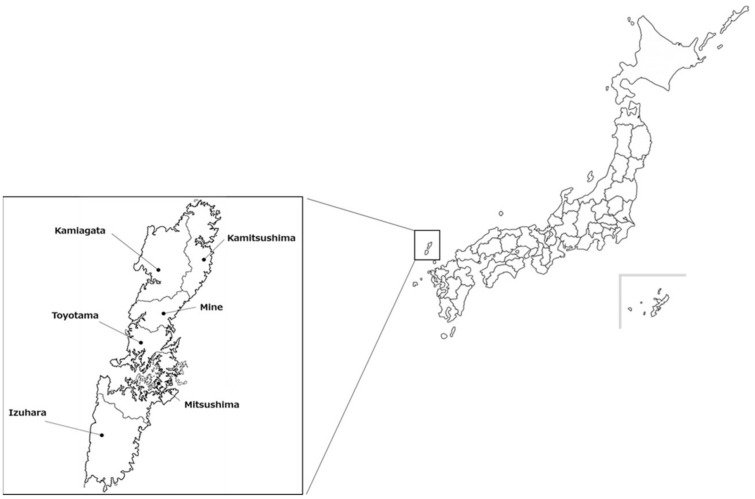
Geographical map of Tsushima Island. Tsushima Island is divided into two main islands: Kamijima to the north and Shimojima to the south. Kamijima is composed of four boroughs (Kamitsushima, Kamiagata, Mine, and Toyotama), and Shimojima has two boroughs (Mitsushima and Izuhara). The Tsushima Leopard Cat Wild Animal Acclimatization Station is located at Izuhara.

Tsushima Island is a part of Nagasaki Prefecture, which is located between the Tsushima Strait and Korea Strait, between Kyushu and the Korean Peninsula. Tsushima Island is divided into two main islands, Kamijima to the north and Shimojima to the south (Figure 1). Tsushima is a habitat for the island’s endemic Tsushima leopard cat (*Prionailurus bengalensis euptilurus*), which is an endangered species actively protected by the Ministry of the Environment of Japan (https://ikilog.biodic.go.jp/Rdb/booklist). Infections in Tsushima leopard cats by pathogens including *Bartonella clarridgeiae*, *Anaplasma bovis*, *Hepatozoon felis*, and feline immunodeficiency virus have been demonstrated in previous studies [21,22,23,24]. Some pathogens can be transmitted to Tsushima leopard cats from both domestic and wild animals. The invasion and spread of SFTSV across Tsushima Island could threaten not only humans but also carnivore species such as cats, dogs, and Tsushima leopard cats. A recent investigation reported that the tick species *Haemaphysalis longicornis* and *Amblyomma testudinarium* act as vectors for SFTSV distributed on Tsushima Island [25]. Therefore, we conducted a serological surveillance study of SFTSV infection in wild and companion animals on Tsushima Island.

## 2. Materials and Methods

All serum samples were collected from animals throughout Tsushima Island. A total of 323 and 84 samples were collected from domestic cats and dogs, respectively, between 2013 and 2022. These animals were domestically owned and visited animal hospitals for neutralization or general medical care. A total of 50 and 71 samples were collected from wild boars and Sika deer, respectively, which were captured by professional hunters using corral traps between 2015 and 2020. Six samples were collected from Tsushima leopard cats at the Tsushima Leopard Cat Wild Acclimatization Station in the southern part of the island (Figure 1). Three of these cats were transferred from zoos outside the island to the center and bred at the center for more than two months, whereas two cats were rescued in the wild on the island. Collectively, these five animals were trained to adapt to a semi-wild environment. One Tsushima leopard cat, MT105, was captured because it had entered the center from a nearby field. No clinical abnormalities were noted at the time of blood sampling. During blood sampling from the animals, standard infection controls including needle stick injury prevention, hand hygiene, and wearing gloves were performed. Animal serum samples were stored at −80°C until use. Animal samples were collected under the guidelines regulating animal use and ethics at Kagoshima University (permission number: VM20035).

To detect anti-SFTSV antibodies, serum samples from domestic dogs, cats, and Tsushima leopard cats were tested for SFTS-specific IgG and IgM using an indirect ELISA, as described previously with a minor modification [11,26]. Antibodies against feline IgG and IgM were used for serum from domestic cats and Tsushima leopard cats. Because the sensitivity and specificity of indirect ELISA are different among animal species, cut-off values of optimal density (OD) for each animal species were determined (our unpublished data). Cut-off values for IgG were determined by comparison with the virus-neutralization test as follows: 0.65 for dogs and 0.74 for domestic cats. Cut-off values for IgM were determined by a comparison between SFTS and non-SFTS animals, with 0.41 for dogs and 0.58 for domestic cats. For the Tsushima leopard cat, a cut-off value of 0.74 for IgG and 0.58 for IgM were tentatively applied. For analysis of serum samples from wild boars and Sika deer, protein A/G was used as the secondary antibody [14], and the cut-off OD values were 0.16 and 0.39 for wild boars and Sika deer, respectively. For ELISA-positive serum samples, the 50% focus reduction neutralization test (FRNT_50_) was performed using Vero cells (ATCC^®^ CCL-81™) and SFTSV according to previously reported studies [26,27]. SFTSV HB29 was kindly provided by Dr. Xin Li and Dr. MiFang Liang of the Chinese CDC. In this study, all animal samples were handled at Biosafety level (BSL)-2. The virus-neutralization test was conducted in the BSL-3 facilities at the National Institute of Infectious Diseases, Japan.

## 3. Results

### 3.1. Seroprevalence in the Animals

The seroprevalence of SFTSV infection in the animals examined in this study is summarized in Table 1, and the OD values for ELISA and FRNT50 in seropositive animals are summarized in Table 2. In this study, 2 of 84 dogs (2.4%), 7 of 323 domestic cats (2.2%), 1 of 50 wild boars (2.0%), and 1 of 6 Tsushima leopard cats (16.7%) harbored virus-neutralizing antibodies against SFTSV. All seropositive domestic dogs and cats had outdoor access. Although many anti-SFTSV antibody-positive animals were of unknown age, they were presumed to be adults from their body weight. There was no significant correlation between sex and seroprevalence. No Sika deer harbored antibodies against SFTSV. No IgM-positive dogs or cats were identified. 

### 3.2. Information on Positive Years and Areas

**Figure 2:**
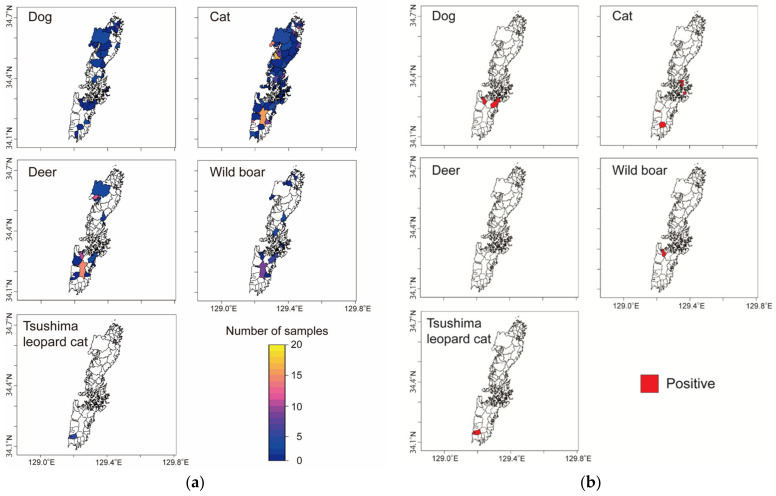
Sites at which animal samples were collected (**a**) and locations of SFTSV-positive animals (**b**), depicted using a geographical information system (GIS) based on geo-coordinates using QGIS software, version 2.12.3 (QGIS.org, QGIS Geographic Information System. QGIS Association; http://www.qgis.org, 2021) and R statistical software (R version 4.2.1., R Core Team 2021).

Except for one seropositive domestic cat sample collected in 2013, all seropositive animal samples were collected after 2019 (Table 3). Distributions of animals examined in this study and anti-SFTSV antibody-positive animals were visualized on a map using a geographical information system (R software ver. 4.2.1 and QGIS software ver. 3.22.8) (Figure 2). Although serum samples were collected from all over Tsushima Island, anti-SFTSV antibody-positive animals were found in only two areas: Mitsushima and Izuhara (Table 3).

## 4. Discussion

Two human patients with SFTS were identified on Tsushima Island in 2013 and 2017 (https://www.pref.nagasaki.jp/); to the best of our knowledge, there were no reports of animals with symptoms of SFTSV infection on this island until now. In the present study, the seroprevalence of animals on Tsushima Island was examined to characterize the spread of SFTSV in wild and companion animals. In a previous study by Hayasaka et al. [28], 50 samples from wild boars collected between 2006 and 2012 on Tsushima Island were investigated, but no seropositive animals were found. In the present study, samples from 10 of 11 SFTSV-positive animal samples were collected after 2019, while one sample was collected in 2013. This suggests that SFTSV might have presented at least once in 2013 and has only recently spread in the environment on the island. However, the seropositivity ratios for Sika deer and wild boars on Tsushima Island in our present study were markedly lower than ratios for other parts of western Japan [17,18,19,20], indicating low circulation of SFTSV among wild animals on Tsushima Island until now.

The estimated number of Tsushima leopard cats in the wild is less than one hundred [29] and we succeeded in the analysis of six serum samples from this endangered species in this study. Among them, we identified one SFTSV-positive Tsushima leopard cat, which was captured at the Tsushima Leopard Cat Wild Acclimatization Station in the southern part of Tsushima Island, and this information was released to the press by the Ministry of the Environment in July 2022 (https://kyushu.env.go.jp/press_00003.html). SFTSV had already affected Tsushima leopard cats, and this positive leopard cat had survived SFTSV infection. Tsushima leopard cats primarily inhabit the northern part of Tsushima Island, and few of them are found in the southern area [20]. Because all 11 seropositive animals in this study were found in the southern part of the island, the SFTSV distribution appears to be limited to date. In Japan, many SFTS cats have been identified since 2017, and their fatality rate is as high as 67% [13]. Lethal SFTS in two captive cheetahs were also recognized [10], and *Felidae* seems to be sensitive to SFTSV infection. Because SFTS could be a threat to Tsushima leopard cats, active monitoring of the spread of the virus and countermeasures to protect Tsushima leopard cats from SFTSV infection will be necessary.

Investigations of SFTSV infection among wildlife are important for indirectly assessing the risk of infection in humans and other animals [17]. To monitor the risk of SFTSV in the future, continuous surveillance of the virus distribution is needed. Unfortunately, the virus could not be detected in this study. Because the features of this virus, including its pathogenicity, are important for understanding SFTSV infection among animals on Tsushima Island, further studies focusing on virus detection and isolation should be performed.

In conclusion, we found animals positive for SFTSV infection on Tsushima Island for the first time. SFTSV might have only recently spread in the environment and the antibody-positive animals were distributed in a limited area on the island. These results indicate an increased risk of SFTSV infection for humans and animals, including endangered Tsushima leopard cats, and countermeasures to prevent the spread of SFTSV on Tsushima Island are required. To protect the endangered Tsushima leopard cats from SFTSV infection, the One Health approach should be conducted by multiple sectors.

## Figures and Tables

**Table 1:** Seroprevalence of SFTSV infection in animals on Tsushima Island.

Animal Species	Parameter		Total Number	Antibody-Positive	
				Number	(%)
**Domestic dogs**			**84**	**2**	**(2.4)**
	Agegroup	<1 year	2	0	(0)
		1–5 years	20	0	(0)
		6–10 years	25	0	(0)
		>11 years	29	1	(3.4)
		Unknown	8	1	(12.5)
	Sex	Male	46	1	(2.2)
		Female	38	1	(2.6)
**Domestic cats**			**323**	**7**	**(2.2)**
	Age group	<1 year	54	0	(0)
		1–5 years	69	1	(1.4)
		6–10 years	6	0	(0)
		>11 years	3	0	(0)
		Unknown	191	6	(3.2)
	Sex	Male	161	3	(1.9)
		Female	162	4	(2.4)
**Wild boars**			**50**	**1**	**(1.8)**
	Body weight	11–30 kg	22	0	(0)
		31–50 kg	24	1	(4.2)
		51–60 kg	3	0	(0)
		Unknown	1	0	(0)
	Sex	Male	41	1	(2.4)
		Female	9	0	(0)
**Sika deer**			**71**	**0**	**(0)**
	Body weight	21–30 kg	7	0	(0)
		31–50 kg	42	0	(0)
		51–60 kg	5	0	(0)
		Unknown	17	0	(0)
	Sex	Male	41	0	(0)
		Female	16	0	(0)
		Unknown	14	0	(0)
**Tsushima leopard cats**			**6**	**1**	**(16.7)**
	Age group	1 year	2	0	(0)
		13 years	1	0	(0)
		Unknown	3	1	(33.3)
	Sex	Male	3	1	(33.3)
		Female	3	0	(0)

**Table 2:** Information regarding SFTSV-positive animals.

Animal Species	ID	Sex	Age (year)	Living Environment	Sample Collection	ELISA (OD)		FRNT_50_
						IgG	IgM	
Domestic dog	223	Male	Unknown	Outdoor	Jul-19	0.96	0.00	≥1:160
Domestic dog	427	Female	12	Outdoor and indoor	Dec-21	1.83	0.17	≥1:160
Domestic cat	14	Spayed	Unknown	Outdoor	Dec-13	1.12	−0.05	1:10
Domestic cat	244	Spayed	Unknown	Outdoor	Jan-20	2.46	−0.03	≥1:10
Domestic cat	271	Casted	Unknown	Outdoor	Apr-20	1.58	−0.02	1:10
Domestic cat	280	Casted	Unknown	Outdoor and indoor	May-20	0.98	0.06	1:10
Domestic cat	283	Spayed	Unknown	Outdoor and indoor	May-20	2.23	0.00	1:40
Domestic cat	287	Spayed	Unknown	Outdoor	May-20	2.10	−0.01	1:80
Domestic cat	335	Casted	5	Outdoor	Oct-20	1.35	−0.15	1:40
Wild boar	223	Male	Unknown	Wildlife	Jul-19	0.24 *	-	≥1:160
Tsushima leopard cat	MT105 †	Male	Unknown	Wildlife	May-22	1.23	0.03	≥1:10

* OD value determined by ELISA using protein A/G as the secondary antibody. † Leopard cat was captured after entering the Tsushima Leopard Cat Wild Acclimatization Station from the field.

**Table 3:** Seroprevalence of SFTSV infection in animals by year and area.

Year/Area		Positive Number/Tested Number (%)									
		Domestic Dogs		Domestic Cats		Wild Boars		Sika Deer		Tsushima Leopard Cats	
**Year**	2013	-		1/13	(7.7)	-*		-		-	
	2014	0/10	(0)	0/39	(0)	-		-		-	
	2015	0/6	(0)	0/35	(0)	0/1	(0)	-		-	
	2016	0/2	(0)	0/20	(0)	-		-		-	
	2017	-		0/7	(0)	-		-		-	
	2018	0/5	(0)	0/13	(0)	-		0/2	(0)	-	
	2019	1/15	(6.7)	0/46	(0)	1/49	(2)	0/63	(0)	-	
	2020	0/29	(0)	6/83	(7.2)	-		0/6	(0)	-	
	2021	1/16	(6.3)	0/61	(0)	-		-		0/4	(0)
	2022	0/1	(0)	0/5	(0)	-		-		1/2	(50)
**Area**	Kamitsushima	0/24	(0)	0/46	(0)	0/2	(0)	-		-	
	Kamiagata	0/20	(0)	0/62	(0)	0/3	(0)	0/20	(0)	-	
	Mine	0/4	(0)	0/20	(0)	0/4	(0)	0/3	(0)	-	
	Toyotama	0/5	(0)	0/47	(0)	0/5	(0)	-		-	
	Mitsushima	2/17	(11.8)	3/63	(4.8)	1/19	(5.3)	0/18	(0)	-	
	Izuhara	0/14	(0)	4/84	(4.8)	0/17	(0)	0/30	(0)	1/6	(16.7)
Total		2/84	(2.4)	7/322	(2.2)	1/50	(1.8)	0/71	(0)	1/6	(16.7)

* Not tested.

## Data Availability

Not applicable.
